# Leucine-rich alpha-2 glycoprotein in combination with C-reactive protein for predicting endoscopic activity in Crohn’s disease: a single-centre, cross-sectional study

**DOI:** 10.1080/07853890.2025.2453083

**Published:** 2025-01-17

**Authors:** Yoshiaki Takada, Hiroki Kiyohara, Yohei Mikami, Masataka Taguri, Ryoya Sakakibara, Yasuhiro Aoki, Kosaku Nanki, Takaaki Kawaguchi, Yusuke Yoshimatsu, Shinya Sugimoto, Tomohisa Sujino, Kaoru Takabayashi, Naoki Hosoe, Haruhiko Ogata, Motohiko Kato, Yasushi Iwao, Nobuhiro Nakamoto, Takanori Kanai

**Affiliations:** a Department of Internal Medicine, Division of Gastroenterology and Hepatology, Keio University School of Medicine, Tokyo, Japan; b Department of Health Data Science, Tokyo Medical University, Tokyo, Japan; c Center for Diagnostic and Therapeutic Endoscopy, Keio University School of Medicine, Tokyo, Japan; d Center for Preventive Medicine, Keio University, Tokyo, Japan

**Keywords:** Crohn’s disease, leucine-rich alpha-2 glycoprotein, C-reactive protein, combination, specificity

## Abstract

**Background and objective:**

Leucine-rich alpha-2 glycoprotein (LRG) is a novel biomarker for Crohn’s disease (CD). The utility of combination use of LRG and C-reactive protein (CRP) has not been reported. This study aimed to investigate the diagnostic performance of LRG in combination with CRP to predict endoscopic activity.

**Methods:**

A single-centre, retrospective, cross-sectional study was conducted. Patients with CD who had serum LRG concentrations measured at least once between June 2020 and May 2021 were enrolled. Clinical activity was evaluated with the Harvey–Bradshaw Index (HBI). Spearman’s rank correlation coefficient (*r_s_*) was used to analyse the correlations between the HBI, LRG concentrations and CRP concentrations. In patients undergoing ileocolonoscopy or balloon-assisted enteroscopy within 60 days before or after LRG measurement, endoscopic activity was evaluated with the simple endoscopic score for Crohn’s disease (SES-CD). The diagnostic performance of LRG and CRP for endoscopic activity was evaluated using receiver operating characteristic (ROC) analysis.

**Results:**

Four hundred and eighty-nine measurements in 343 patients were analysed. Although a strong correlation was found between LRG and CRP concentrations (*r_s_* = 0.75), the HBI did not well correlate with LRG or CRP concentrations. Endoscopic activity was analysed in 56 patients. In diagnosing endoscopically moderate to severe activity (SES-CD > 6), the area under the ROC curve of LRG was greater than that of CRP (0.74 vs. 0.63; *p* = .037). The optimal cut-off value estimated by Youden’s index was 15.5 µg/mL for LRG, and 0.13 mg/dL for CRP. LRG and CRP concentrations were considered positive when they were above these cut-off values, and the sensitivity and specificity for an SES-CD > 6 were 58.3% and 93.8%, respectively. Dual positivity of LRG and CRP showed the highest specificity.

**Conclusions:**

Combination use of dual positive LRG and CRP is useful for diagnosing endoscopically moderate to severe disease.

## KEY MESSAGES

Serum leucine-rich alpha-2 glycoprotein (LRG) is useful for predicting endoscopically moderate to severe Crohn’s disease when used in combination with C-reactive protein (CRP). Leucine-rich alpha-2 glycoprotein and CRP have high specificity for predicting endoscopically moderate to severe activity. Therefore, therapeutic adjustment for patients with definitive high LRG and CRP concentrations may be considered even in the absence of endoscopic evaluation.

## Introduction

The Selecting Therapeutic Targets in Inflammatory Bowel Disease (STRIDE)-II has been proposed as a treat-to-target strategy for inflammatory bowel disease (IBD) [1]. In this strategy, short-term targets include symptomatic remission and normalization of serum C-reactive protein (CRP) concentrations, intermediate targets include normalization of faecal calprotectin, and long-term targets include endoscopic healing and normalized quality of life. Faecal calprotectin is widely accepted as a noninvasive biomarker in patients with IBD. However, the stool calprotectin test has some disadvantages, such as the complexity of collecting and bringing stool samples, the difficulty in on-demand sampling, and sample instability [2].

Recently, serum leucine-rich alpha-2 glycoprotein (LRG), a 50 kDa glycoprotein, has attracted attention as a novel biomarker in IBD [3,4]. CRP is produced through the interleukin (IL)-6-mediated pathway, whereas LRG is produced by inflammatory cytokines other than IL-6, including IL-1β, IL-22 and tumour necrosis factor-α [3]. Therefore, serum LRG concentrations may be a useful biomarker to detect endoscopic activity even in cases where CRP concentrations are not elevated. Although the usefulness of serum LRG concentrations in treating IBD has been reported [2,4–14], its usefulness as a biomarker has not been well established. In particular, the usefulness of LRG in combination with CRP has not been fully clarified.

This study aimed to investigate the diagnostic accuracy of serum LRG concentrations in patients with Crohn’s disease (CD), and to evaluate the utility of combination use of LRG and CRP for predicting endoscopic activity.

## Materials and methods

### Study design and patients

A retrospective, cross-sectional study was conducted at Keio University Hospital. Patients with CD in whom serum LRG concentrations were measured at least once at our institution between 1 June 2020 and 31 May 2021 were included in this study. The diagnosis of CD was made on the basis of established diagnostic criteria [15]. Patients in whom sufficient information was not available to assess clinical activity based on the Harvey–Bradshaw Index (HBI) were excluded.

### Assessment of disease activity and biomarkers

Clinical activity was assessed using the HBI. An HBI score < 4 points was defined as clinical remission [16]. Active disease was defined as an HBI ≥ 4 points. Endoscopic activity was assessed in patients who underwent ileocolonoscopy or balloon-assisted enteroscopy within 60 days before or after the measurement of serum LRG concentrations. Under the Japanese health insurance system, reimbursement is not permitted if LRG measurement is performed at the same month as an endoscopy. Consequently, because this was a retrospective observational study, serum LRG concentrations were measured in a different month to when endoscopy was performed, in principle. Blood tests were usually carried out in conjunction with outpatient consultations and the interval between hospital visits often exceeded 2 months. Therefore, we allowed the interval between LRG measurement and endoscopy to be up to 60 days. Endoscopic activity was assessed with the simple endoscopic score for Crohn’s disease (SES-CD) [17]. The definition of endoscopic activity based on the SES-CD in this study was as follows: endoscopic remission, an SES-CD ≤ 2 points; mild disease, an SES-CD > 2 and ≤ 6 points; moderate to severe disease, an SES-CD > 6 points [18,19]. All endoscopists were experts in IBD and were well trained in using the SES-CD. Two IBD experts assessed images of endoscopy independently and assigned SES-CDs for each examination. In case of discrepancies in the evaluation, the findings were discussed and, if necessary, the opinion of another IBD expert was consulted. Serum LRG and CRP concentrations were evaluated as biomarkers, and other laboratory data, such as haemoglobin concentrations, the white blood cell count, serum albumin concentrations, total cholesterol concentrations and the erythrocyte sedimentation rate, were also examined.

### Endpoint

The endpoint of this study was the diagnostic performance of serum LRG and CRP concentrations for clinical and endoscopic activity at the time of the laboratory examination or endoscopy. The diagnostic performance of LRG and CRP was evaluated independently, and was also investigated in combination with each other.

### Statistical analyses

Data are shown as the median (interquartile range (IQR)) for continuous variables, and the number (percentage) for categorical variables. Categorical variables were compared using Fisher’s exact test, and numerical variables were compared with the Mann–Whitney *U*-test for two groups or the Kruskal–Wallis test for three groups. Multiple testing among the three groups with significant differences in the Kruskal–Wallis test was corrected by Dunn’s test. Correlations between the HBI, CRP concentrations and LRG concentrations were evaluated using Spearman’s rank correlation coefficient (*r_s_*). A receiver operating characteristic (ROC) analysis was conducted to evaluate the diagnostic performance of LRG or CRP regarding clinical or endoscopic activity. In the ROC analysis, the area under the curve (AUC) was calculated and the optimal cut-off value was determined using Youden’s index. Delong’s test was used to compare the AUCs. In case of multiple LRG measurements in the same patient during the observation period, each observation point was treated as independent. A *p* value < .05 was considered statistically significant and all tests were two-sided. Statistical analyses were performed using JMP 16 software (SAS Institute Japan Ltd., Tokyo, Japan) or Stata version 17 (StataCorp, College Station, TX).

### Ethical considerations

This study was approved by the Ethics Committee of Keio University School of Medicine (approval number: 20150210). Written informed consent was not required, but an opt-out was implemented to provide patients with the opportunity to express their disagreement with participation in this study, in accordance with local and national guidelines. This manner is justified by the Japanese ethical guidelines (available in Japanese only) and has been approved by the Ethics Committee of Keio University School of Medicine. The study was conducted in accordance with the ethical standards set forth in the 1964 Declaration of Helsinki and its later amendments.

## Results

### Study population and patients’ characteristics

**Figure 1.**
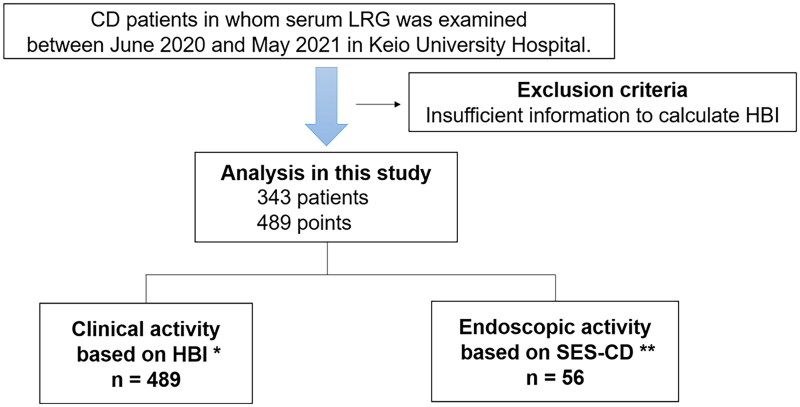
Study diagram. A total of 489 measurements of serum LRG concentrations in 343 patients were included in the primary analysis. Endoscopy was performed in 56 patients within 60 days before or after measuring LRG concentrations. *Clinical remission, HBI < 4; active disease, HBI ≥ 4. **Endoscopic remission, an SES-CD ≤ 2; mild disease, an SES-CD >2 and ≤6; and moderate to severe disease, an SES-CD > 6. HBI: Harvey–Bradshaw Index; SES-CD: simple endoscopic score for Crohn’s disease.

Serum LRG concentrations were measured at 489 points in 343 patients with CD during the observational period after the exclusion of patients with insufficient information (Figure 1). Endoscopy was performed in 56 patients within 60 days before or after the measurement of LRG concentrations. The patients’ characteristics are shown in Table 1.

**Table 1.** Patients’ characteristics.

Characteristics	Total (*n* = 489)	Clinical remission (*n* = 370)	Active disease (*n* = 119)	*p* Value
Male sex, *n* (%)	328 (67.1)	240 (64.9)	88 (74.0)	.173^a^
Age, years, median (IQR)	38 (31–48)	37 (30–46)	43 (32–50)	.001^b^
Disease location, *n* (%)				.140^a^
L1	83 (17.0)	69 (18.7)	14 (11.8)	
L2	50 (10.2)	40 (10.8)	10 (8.4)	
L3	356 (72.8)	261 (70.5)	95 (79.8)	
Harvey–Bradshaw Index	1 (0–3)	1 (0–2)	6 (4–7)	<.001^b^
Current or previous treatment, *n* (%)				
Steroid	92 (18.8)	52 (14.1)	40 (33.6)	<.001^a^
Thiopurine	220 (45.0)	171 (46.2)	49 (41.2)	.343^a^
Anti-TNF-α	344 (70.4)	265 (71.6)	79 (66.4)	.300^a^
Vedolizumab	11 (2.3)	6 (1.6)	5 (4.2)	.147^a^
Ustekinumab	64 (13.1)	40 (10.8)	24 (20.2)	.012^a^
Laboratory data, median (IQR)				
LRG, µg/mL	13.3 (10.5–18.5)	12.3 (10.1–16.7)	16.7 (12.2–24.0)	<.001^b^
CRP, mg/dL	0.09 (0.03–0.28)	0.07 (0.03–0.23)	0.18 (0.06–0.54)	<.001^b^
WBCs, /µL	5900 (5000–7250)	5800 (4900–7000)	6500 (5400–7800)	<.001^b^
Haemoglobin, g/dL	13.9 (12.6–15.1)	14.0 (12.7–15.1)	13.6 (12.3–14.9)	.069^b^
Platelets, ×10^4^/µL	26.3 (22.2–31.3)	26.3 (22.2–30.8)	26.2 (22.0–32.5)	.571^b^
Albumin, g/dL	4.1 (3.8–4.4)	4.2 (3.9–4.4)	4.0 (3.8–4.2)	<.001^b^
Total cholesterol, mg/dL	166 (143–192)	169 (148–193)	151 (127–181)	<.001^b^

CRP: C-reactive protein; IQR: interquartile range; LRG: leucine-rich alpha-2 glycoprotein; TNF: tumour necrosis factor; WBC: white blood cells.

Data are shown as the number (%) or median (IQR). The disease location was classified according to the Montreal classification: L1: ileal disease; L2: colonic disease; L3: ileocolonic disease. Characteristics between the groups of clinical remission and active disease were compared and analysed with ^a^Fisher’s exact test or the ^b^Mann–Whitney *U*-test.

### Diagnostic performance of LRG and CRP for clinical activity

**Figure 2.**
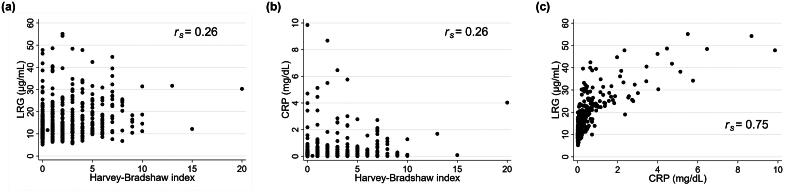
Correlations between clinical activity, LRG concentrations and CRP concentrations. Spearman’s rank correlation coefficients between (a) the HBI and LRG concentrations, (b) the HBI and CRP concentrations and (c) LRG and CRP concentrations were calculated. *r_s_*: Spearman’s rank correlation coefficient; CRP: C-reactive protein; HBI: Harvey–Bradshaw Index; LRG: leucine-rich alpha-2 glycoprotein.

**Figure 3.**
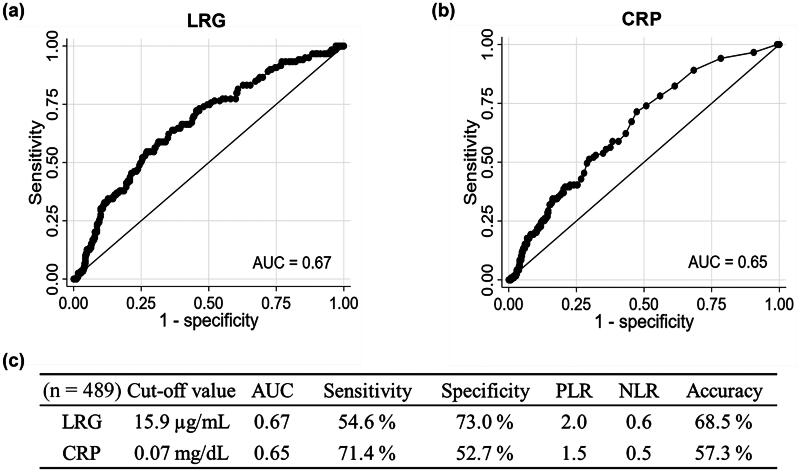
Diagnostic performance of LRG and CRP concentrations for active disease. Receiver operating characteristic curves of (a) LRG concentrations and (b) CRP concentrations for diagnosing active disease. (c) The AUC, sensitivity, specificity, PLR, NLR and accuracy at the optimal cut-off value. The optimal cut-off values of LRG and CRP were estimated using Youden’s index. AUC: area under the curve; CRP: C-reactive protein; LRG: leucine-rich alpha-2 glycoprotein; NLR: negative likelihood ratio; PLR: positive likelihood ratio.

The characteristics of patients in clinical remission (*n* = 370) and in those with active disease (*n* = 119) are shown in Table 1. The median HBI was significantly lower in the clinical remission group than in the active disease group (*p* < .001). The median LRG concentration was significantly lower in the clinical remission group than in the active disease group (*p* < .001). Additionally, the median CRP concentration was significantly lower in the clinical remission group than in the active disease group (*p* < .001). The correlations between the HBI, LRG concentrations and CRP concentrations are shown in Figure 2. The *r_s_* between the HBI and LRG concentrations was 0.26, and it was similar to that between the HBI and CRP concentrations (*r_s_* = 0.26). A strong correlation was found between LRG and CRP concentrations (*r_s_* = 0.75). In the ROC analysis for the diagnostic performance of LRG and CRP concentrations for active disease, the AUC was similar between these two biomarkers (LRG, 0.67; CRP, 0.65; *p* = .339) (Figure 3(a,b)). The sensitivity and specificity were 54.6% and 73.0% for LRG concentrations, and 71.4% and 52.7% for CRP concentrations, respectively (Figure 3(c)).

### Endoscopic activity and biomarkers

Among the 56 patients in whom endoscopy was performed, endoscopic remission (SES-CD ≤ 2), mild disease (SES-CD >2 and ≤6) and moderate to severe disease (SES-CD > 6) were observed in 12, 20 and 24 patients, respectively. The patients’ characteristics in these three groups are shown in Supplementary Table 1. Serum LRG concentrations were significantly different between the three groups (*p* = .007). In the multiple comparison with Dunn’s test, serum LRG concentrations were significantly higher in the moderate to severe disease group than in the endoscopic remission group (*p* = .032) and in the mild disease group (*p* = .001). LRG concentrations were not different between the endoscopic remission and mild disease groups (Supplementary Figure 1). Serum CRP concentrations were not different between the three groups (Supplementary Table 1). Perianal disease and extraintestinal manifestations at the time of LRG measurement were investigated. Active perianal abscess was identified in two patients. Regarding extraintestinal manifestations, joint and skin complications were observed in one and three patients, respectively. No other infectious diseases were identified. Details on these cases are shown in Supplementary Table 2.

### Diagnostic performance of LRG and CRP for endoscopic activity

**Figure 4.**
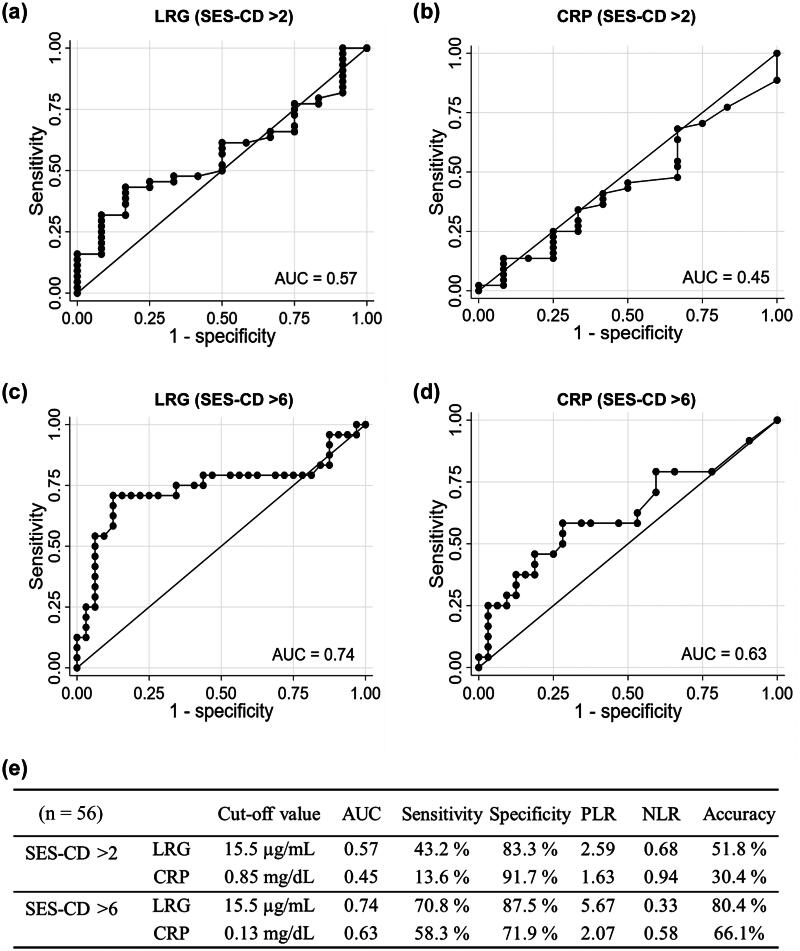
Diagnostic performance of LRG and CRP concentrations for endoscopic activity. Receiver operating characteristic curves of (a) LRG for an SES-CD > 2, (b) CRP for an SES-CD > 2, (c) LRG for an SES-CD > 6 and (d) CRP for an SES-CD > 6. (e) The AUC, sensitivity, specificity, PLR, NLR and accuracy at the optimal cut-off value. The optimal cut-off values were estimated using Youden’s index. AUC: area under the curve; CRP: C-reactive protein; LRG: leucine-rich alpha-2 glycoprotein; NLR: negative likelihood ratio; PLR: positive likelihood ratio; SES-CD: simple endoscopic score for Crohn’s disease.

Two different thresholds of the SES-CD were used for the ROC analysis to investigate the diagnostic performance of LRG and CRP for endoscopic activity: an SES-CD > 2 to distinguish endoscopic remission and non-remission, and an SES-CD > 6 to identify endoscopically moderate to severe disease. In diagnosing an SES-CD > 2, the AUCs of LRG and CRP concentrations were 0.57 and 0.45, respectively (Figure 4(a,b)). Regarding the diagnosis of moderate to severe disease, the AUC for LRG concentrations was 0.74 with an optimal cut-off value of 15.5 µg/mL (sensitivity, 70.8%; specificity, 87.5%), whereas the AUC for CRP concentrations was 0.63 with an optimal cut-off value of 0.13 mg/dL (sensitivity, 58.3%; specificity, 71.9%) (Figure 4(c–e)). The AUC was significantly higher for LRG concentrations than for CRP concentrations (*p* = .037).

### Combination use of LRG and CRP for detecting endoscopically moderate to severe disease

We next investigated whether the combination of LRG and CRP is useful in diagnosing endoscopically moderate to severe (SES-CD > 6) cases. The same cut-off values as those calculated in the ROC analysis with Youden’s index (LRG, 15.5 µg/mL; CRP, 0.13 mg/dL) were used. Of the 56 patients in whom endoscopy was performed, 28, 7, 5 and 16 were in the groups of negative (concentrations were below the cut-off value) for both CRP and LRG, positive (concentrations were above the cut-off value) only for CRP, positive only for LRG and positive for both CRP and LRG, respectively. Because of the small number of patients in whom LRG or CRP alone was positive, we conducted an analysis in the following three groups: both negative (double negative, DN (*n* = 28)), LRG or CRP was positive (single positive, SP (*n* = 12)) and both positive (double positive, DP (*n* = 16)). Moderate to severe disease was present in 25.0% (7/28), 25.0% (3/12) and 87.5% (14/16) of patients in the DN, SP and DP groups, respectively (*p* < .001). In a univariable logistic regression analysis, DP was predictive for moderate to severe disease, whereas SP was not (DN was used as the reference; Supplementary Table 3). We next analysed the diagnostic accuracy of (i) LRG- and/or CRP-positive (i.e. SP or DP) and (ii) both LRG- and CRP-positive (i.e. DP) for endoscopically moderate to severe disease. The diagnostic performance of LRG in combination with CRP for an SES-CD > 6 was evaluated (Table 2). The sensitivity and specificity were 70.8% and 65.6%, respectively, if positive was defined as at least one of LRG or CRP concentrations being above the cut-off level (i.e. LRG- and/or CRP-positive). In this setting, the positive likelihood ratio (PLR) was 2.06, which was not higher than that in the analysis of LRG alone (Figure 4(e)). The sensitivity and specificity were 58.3% and 93.8%, respectively, if positive was defined as both LRG and CRP concentrations being above the cut-off level. Under this definition, the PLR was 9.33.

**Table 2.** Diagnostic performance of LRG in combination with CRP for endoscopically moderate to severe disease.

SES-CD > 6 (*n* = 56)		Cut-off value	Sensitivity	Specificity	PLR	NLR	Accuracy
Both LRG (+) and CRP (+)	LRG	15.5 µg/mL	58.3%	93.8%	9.33	0.44	78.6%
	CRP	0.13 mg/dL					
LRG (+) and/or CRP (+)	LRG	15.5 µg/mL	70.8%	65.6%	2.06	0.44	65.5%
	CRP	0.13 mg/dL					

CRP: C-reactive protein; LRG: leucine-rich alpha-2 glycoprotein; NLR: negative likelihood ratio; PLR: positive likelihood ratio; SES-CD: simple endoscopic score for Crohn’s disease.

Serum LRG and CRP concentrations were classified as positive or negative on the basis of optimal cut-off values for an SES-CD > 6 in the receiver operating characteristic analysis as shown in Figure 4(e). The diagnostic performance was evaluated in the following two settings: (i) both LRG- and CRP-positive and (ii) LRG- and/or CRP-positive (i.e. at least one of LRG or CRP was positive).

### Prediction of endoscopically moderate to severe disease using dual positive LRG and CPR in clinical remission and active disease

The diagnostic utility of dual positive LRG and CRP for predicting endoscopically moderate to severe disease was also investigated in the subpopulation of clinical remission and active disease, independently (Table 3). Among 56 patients with endoscopic evaluation, 36 were in clinical remission. Endoscopically moderate to severe disease (i.e. SES-CD > 6) was observed in 14/36 (38.9%) patients. Six of 36 (16.7%) patients were classified as dual positive. Among those in clinical remission, the sensitivity and specificity for an SES-CD > 6 was 26.7% and 95.5%, respectively, and the PLR was 5.93. In the remaining 20 patients with active disease, an SES-CD > 6 was observed in 10 (50.0%) patients, and dual positive was observed in nine (45.0%) patients. The sensitivity and specificity for an SES-CD > 6 in the subpopulation with active disease were 80.0% and 90.0%, respectively, and the PLR was 8.00.

**Table 3.** Diagnostic performance of dual positive LRG and CRP for endoscopically moderate to severe disease in clinical remission and active disease.

SES-CD > 6		Cut-off value	Sensitivity	Specificity	PLR	NLR	Accuracy
Both LRG (+) and CRP (+)	Clinical remission (*n* = 36)	LRG: 15.5 µg/mL	26.7%	95.5%	5.93	0.77	72.2%
		CRP: 0.13 mg/dL					
	Active disease (*n* = 20)	LRG: 15.5 µg/mL	80.0%	90.0%	8.00	0.22	85.0%
		CRP: 0.13 mg/dL					

CRP: C-reactive protein; LRG: leucine-rich alpha-2 glycoprotein; NLR: negative likelihood ratio; PLR: positive likelihood ratio; SES-CD: simple endoscopic score for Crohn’s disease.

LRG and CRP were classified as positive when both LRG and CRP concentrations were above the optimal cut-off values for an SES-CD > 6 in the receiver operating characteristic analysis as shown in Figure 4(e). If LRG or CRP concentrations were not above the cut-off value, this was classified as negative.

## Discussion

In the present study, the diagnostic performances of LRG and CRP concentrations in patients with CD were evaluated. Serum LRG concentrations were useful for predicting endoscopically moderate to severe disease (i.e. SES-CD > 6) (Figure 4(e)). Furthermore, dual positive LRG and CRP showed a high specificity and PLR in diagnosing endoscopically moderate to severe disease (Tables 2 and 3).

The novelty of this research is that a combination of high LRG and CRP concentrations may predict endoscopically moderate to severe activity, which is likely to require therapeutic intervention, with high specificity, potentially preceding endoscopic evaluation. The usefulness of LRG in predicting disease activity of CD has been reported in previous studies and it has also been compared with CRP [2,6,7,10,14,20]. However, to the best of our knowledge, the diagnostic performance of LRG in combination with CRP has not been reported. In the present study, we evaluated the sensitivity, specificity, PLR, negative likelihood ratio and accuracy in predicting endoscopically moderate to severe disease (i.e. SES-CD > 6) with the following two definitions: (i) both LRG and CRP concentrations above the cut-off value were classified as positive and (ii) LRG and/or CRP concentrations above the cut-off value were classified as positive. Consequently, the highest specificity (93.8%) and PLR (9.33) were observed when both LRG and CRP concentrations above the cut-off value were considered positive (Table 2). Therefore, endoscopically moderate to severe disease is highly suspected when both LRG and CRP concentrations are elevated. Furthermore, when LRG and/or CRP concentrations higher than the cut-off level were considered positive, the sensitivity for diagnosing an SES-CD > 6 was not superior to that of LRG alone (Figure 4(e), Table 2). This finding could be attributed to the fact that the sensitivity of CRP for diagnosing an SES-CD > 6 was lower than that of LRG (58.3% vs. 70.8%, Figure 4(e)), and that CRP concentrations might be less likely to increase when LRG concentrations are normal in patients with endoscopically moderate to severe disease. In a study conducted by Kawamoto et al., the sensitivity of CRP for predicting intestinal ulcers was lower than that of LRG at the optimal cut-off value (40% vs. 79%), which is consistent with our result [6]. Similar results were obtained in other studies [2,7]. The finding that LRG showed a higher sensitivity to predict moderate to severe disease than CRP is expected because LRG can be induced via IL-6-dependent or IL-6-independent pathways, whereas IL-6 is indispensable for CRP production [3]. To the best of our knowledge, this is the first study to evaluate the diagnostic utility of combination use of LRG and CRP in patients with CD.

When both LRG and CRP concentrations are elevated, there is likely to be involvement of IL-6, which is a common inducer of LRG and CRP [3], in the disease activity. A study showed that serum IL-6 concentrations were predictive for endoscopically moderate to severe activity (SES-CD > 7) in CD (AUC, 0.77) [21]. Therefore, not only LRG but also CRP concentrations may be elevated along with IL-6 production and function in endoscopically moderate to severe disease. Furthermore, a previous study on a mouse model of collagen-induced arthritis suggested that LRG upregulates IL-6 receptor expression in naïve CD4 T cells and also promotes T helper 17 cell differentiation [22]. Although this speculation is based on an animal arthritis model study, elevated LRG concentrations in moderate to severe disease itself might promote CRP production via IL-6 and also by T helper 17 cell differentiation. Further research on humans with IBD is required to investigate the actual mechanism.

Normalization of biomarkers was proposed as an intermediate target to be achieved in the STRIDE-II strategy [1]. This strategy recommends considering changing treatment if this target has not been achieved. The concept of therapeutic adjustment based on clinical activity and biomarkers regardless of endoscopic evaluation in CD has been widely accepted. The results of the CALM trial were used as one of the rationales behind justifying this strategy [23]. In this trial, a better outcome (mucosal healing with the absence of deep ulcers at week 48) was observed in patients with intensive therapeutic adjustment following elevated CRP concentrations, an increase in faecal calprotectin concentrations, prednisone use or non-achieved clinical remission than those in whom treatment was escalated on the basis of the clinical activity index alone. A post hoc analysis of the CALM study suggested that the combined use of CRP and faecal calprotectin concentrations improved the predictive ability of endoscopic healing at week 48 more than each biomarker alone [24]. Furthermore, a study reported that the specificity for predicting intestinal ulcers was higher with dual positive LRG and faecal calprotectin than LRG alone [14], whereas the combination of LRG and CRP was not evaluated. When considering therapeutic adjustment, unnecessary escalation or a change in treatment should be avoided. Regarding this issue, evaluating disease activity with a high specificity is important. Although serum CRP concentrations are one of the most commonly used biomarkers, the sensitivity and specificity are insufficient. In recent studies on the diagnostic performance of biomarkers, the specificity of CRP alone for endoscopically active disease in patients with CD ranged from 55% to 89% [5–7,10,13,14,25,26]. Yasutomi et al. conducted sensitivity analyses for outcomes with various endoscopic activities and reported a specificity of CRP for a modified SES-CD > 6 (76%) that was similar to that in our study [5]. This finding did not appear sufficient to consider a therapeutic change without endoscopic evaluation. In the present study, we found that dual positive LRG and CRP predicted endoscopically moderate to severe disease with a high specificity and PLR. Notably, a high PLR of 8.00 was observed in the active disease subpopulation, which already had a high pre-test probability of endoscopic activity (Table 3). Dual positive LRG and CRP in this subpopulation predicts endoscopically moderate to severe disease with further high post-test probability. This finding suggests that therapeutic adjustment for patients who are suspected as having endoscopic activity (e.g. clinically active disease) following dual positive LRG and CRP could be justified regardless of endoscopic evaluation. The present study findings may help to reduce the need for endoscopy, which is invasive and is not always necessary in certain cases.

The present study has some limitations. First, this was a single-centre, retrospective study. Endoscopic activity was assessed in only a portion of patients (*n* = 56). Therefore, there is a possibility of selection bias. The findings in the present study should also be validated in another population. Second, the effect of extraintestinal manifestations, perianal abscess or other infectious disease, which may increase inflammatory biomarkers, on the diagnostic ability of LRG and CRP for endoscopic activity could not be sufficiently assessed. If LRG and/or CRP concentrations were elevated because of these complications in case of endoscopically quiescent disease, the false positive rate would increase. Nevertheless, the effect of these complications appeared to be minimal in the present study. The reason for this lack of effect is because no patients showed an inadequate elevation of serum LRG or CRP concentrations to above the cut-off level, despite endoscopic remission or mild disease (Supplementary Table 2). In fact, high specificity (equal to low false positive rate) (93.8%) of dual positivity with LRG and CRP was revealed in predicting endoscopically moderate to severe disease in the present study. To evaluate bowel inflammation, a faecal biomarker, which was unfortunately unavailable in our study, might be useful. Further studies including a sufficient number of cases with active extraintestinal manifestations or perianal disease are required to investigate the associations between these complications and the diagnostic performance of the biomarkers. Finally, the interval between measurement of LRG concentrations and endoscopy was allowed to be up to 60 days. Of the 56 patients in whom endoscopy was conducted, therapeutic intervention was provided in three patients between endoscopy and a blood test. Furthermore, the treatment had been changed within 3 months before LRG measurement in another four patients in whom the interval between endoscopy and a blood test was longer than 30 days. Therefore, the LRG concentrations might not have accurately reflected the endoscopic activity in these cases.

## Conclusions

LRG is a useful biomarker to predict moderate to severe endoscopic activity in CD. Additionally, the combination use of LRG and CRP is useful, with a high specificity and PLR for an SES-CD > 6 when both of them being above the cut-off level is judged as positive.

## Supplementary Material

Supplementary_Materials.docx

## Data Availability

The data that support the findings of this study are available from the corresponding authors upon reasonable request.
