# Supplementary material for: Leucine-rich alpha-2 glycoprotein in combination with C-reactive protein for predicting endoscopic activity in Crohn’s disease: a single-centre, cross-sectional study
Source: Ann Med. 2025 Jan 17;57(1):2453083. doi: 10.1080/07853890.2025.2453083 (PMC11748989; doi:10.1080/07853890.2025.2453083)
Supplement: Supplementary_Materials.docx [file IANN_A_2453083_SM4688.docx]

**Supplementary Table 1. Endoscopic activity and patients’ characteristics.**

| Characteristics |  | Endoscopic  remission  n = 12 | Mild  disease  n = 20 | Moderate to  severe disease  n = 24 | p value |
| --- | --- | --- | --- | --- | --- |
| Male, n (%) |  | 6 (50.0) | 15 (75.0) | 18 (75.0) | 0.270 ^a^ |
| Age, years, median (IQR) |  | 34 (29–43) | 38 (28–52) | 35 (31–43) | 0.899 ^b^ |
| Disease location, n (%) |  |  |  |  | 0.842 ^a^ |
| L1 |  | 3 (25.0) | 3 (15.0) | 5 (20.8) |  |
| L2 |  | 0 (0) | 2 (10.0) | 1 (4.2) |  |
| L3 |  | 9 (75.0) | 15 (75.0) | 18 (75.0) |  |
| Perianal disease |  |  |  |  | 0.821 ^a^ |
| Anal fistula |  | 4 (33.3) | 7 (35.0) | 5 (20.8) |  |
| Perianal abscess |  | 1 (8.3) | 1 (5.0) | 0 (0) |  |
| Extraintestinal manifestations |  |  |  |  | 0.504 ^a^ |
| Joint |  | 1 (8.3) | 0 (0) | 0 (0) |  |
| Skin |  | 0 (0) | 1 (5.0) | 2 (8.3) |  |
| Eye |  | 0 (0) | 0 (0) | 0 (0) |  |
| Harvey-Bradshaw index  Current or previous treatment, n (%) |  | 2 (1–4) | 3 (1–5) | 2 (1–7) | 0.779 ^b^ |
| Steroid ^c^ |  | 2 (16.7) | 6 (30.0) | 9 (37.5) | 0.490 ^a^ |
| Thiopurine |  | 3 (25.0) | 11 (55.0) | 8 (33.3) | 0.201 ^a^ |
| Anti TNF-α |  | 7 (58.3) | 10 (50.0) | 16 (66.7) | 0.568 ^a^ |
| Vedolizumab |  | 0 (0) | 1 (5.0) | 0 (0) | 0.571 ^a^ |
| Ustekinumab |  | 3 (25.0) | 2 (10.0) | 3 (12.5) | 0.483 ^a^ |
| Laboratory data, median (IQR) |  |  |  |  |  |
| LRG, µg/mL |  | 12.9 (11.1–15.3) | 11.6 (10.4–13.2) | 18.1 (13.0–26.6) | 0.007 ^b^ |
| CRP, mg/dL |  | 0.12 (0.04–0.57) | 0.06 (0.03–0.13) | 0.17 (0.05–0.96) | 0.095 ^b^ |
| WBC, /µL |  | 7150  (5450–8100) | 5650  (4800–6350) | 6400  (5400–7600) | 0.106 ^b^ |
| Hemoglobin, g/dL |  | 14.1 (12.7–15.0) | 14.1 (12.9–15.4) | 14.2 (12.4–14.9) | 0.490 ^b^ |
| Platelet, x10^4^/µL |  | 30.2 (23.3–34.4) | 23.7 (20.2–27.8) | 27.6 (23.4–34.0) | 0.039 ^b^ |
| Albumin, g/dL |  | 4.3 (4.0–4.5) | 4.4 (4.1–4.5) | 4.1 (3.9–4.4) | 0.179 ^b^ |
| Total cholesterol, mg/dL |  | 185 (163–197) | 171 (165–188) | 158 (139–187) | 0.116 ^b^ |

Data are shown as the number (%) or median (IQR). The disease location was classified according to the Montreal classification: L1, ileal disease; L2, colonic disease; and L3, ileocolonic disease. Regarding the perianal disease, it was classified according to the status when LRG was measured.

Characteristics between the three groups were compared and analyzed with ^a^Fisher’s exact test or ^b^the Kruskal–Wallis test.

CRP, C-reactive protein; IQR, interquartile range; LRG, leucine-rich alpha-2 glycoprotein; TNF, tumor necrosis factor; WBCs, white blood cells.

**Supplementary Table 2. Endoscopic activity, serum LRG and CRP in patients with perianal disease or extraintestinal manifestations.**

| Case | Complication | Endoscopic activity | LRG (µg/mL) | CRP (mg/dL) |
| --- | --- | --- | --- | --- |
| #1 | Active perianal abscess | Remission | 12.4 | 0.10 |
| #2 | Active perianal abscess | Mild | 10.5 | 0.03 |
| #3 | Joint manifestation | Remission | 14.1 | 0.02 |
| #4 | Skin manifestation | Mild | 11.1 | 0.06 |
| #5 | Skin manifestation | Moderate to severe | 9.2 | 0.02 |
| #6 | Skin manifestation | Moderate to severe | 22.2 | 0.49 |

CRP, C-reactive protein; LRG, leucine-rich alpha-2 glycoprotein.

**Supplementary Table 3. Univariable logistic regression analysis of predicting endoscopically moderate to severe disease (SES-CD > 6).**

|  | **OR (95% CI)** | **p** |
| --- | --- | --- |
| Both LRG- and CRP-negative (double negative, n = 28) | Reference | – |
| LRG- or CRP-positive (single positive, n = 12) | 1.00 (0.21–4.77) | 1.000 |
| Both LRG- and CRP-positive (double positive, n = 16) | 21.00 (3.80–116.20) | 0.012 |

CI, confidence interval; CRP, C-reactive protein; LRG, leucine-rich alpha-2 glycoprotein; OR, odds ratio.

**

Supplementary Fig. 1. Endoscopic activity and serum LRG concentrations.**

Box plots of serum LRG concentrations in endoscopic remission, mild disease, and moderate to severe disease groups. The Kruskal–Wallis test with correction for multiple comparison by Dunn’s test was conducted.

LRG, leucine-rich alpha-2 glycoprotein; NS, not significant; *p < 0.05.
